# A Web-Based Pharmacogenomics Search Tool for Precision Medicine in Perioperative Care

**DOI:** 10.3390/jpm10030065

**Published:** 2020-07-21

**Authors:** Sara Zarei, Yensea Costas, Gloria Orozco, Michelle Zaydlin, Ali Mirtar, Mohammad Abouali, Cristina Diaz-Marty, Golnoush Akhlaghipour, Pablo Fernandez Altamirano, Anel R. Gonzalez Cardona, Luz E. Reiley, Hooman Mirzakhani

**Affiliations:** 1 Channing Division of Network Medicine, Department of Medicine, Brigham and Women’s Hospital, Boston, MA 02115, USA; 2 Arasila Biotech, San Diego, CA 92130, USA; amirtar@arasila.com; 3 San Juan Bautista School of Medicine, Caguas, PR 00727, USA; yenseace@sanjuanbautista.edu (Y.C.); michellez@sanjuanbautista.edu (M.Z.); pfernandez@sanjuanbautista.edu (P.F.A.); anelgc@sanjuanbautista.edu (A.R.G.C.); lereiley@sanjuanbautista.edu (L.E.R.); 4 Texas Tech University Health Sciences Center, Lubbock, TX 79424, USA; gorozco@uh.edu; 5 Computational Science Research Center, San Diego State University, San Diego, CA 92182, USA; mabouali@gmail.com; 6 Wellstar Kennestone Regional Medical Center, Atlanta, GA 30060, USA; cristinadm@sanjuanbautista.edu; 7 Department of Neurology, University of California, Los Angeles, CA 90095, USA; golnoush.akhlaghi@gmail.com; 8 Harvard Medical School, Boston, MA 02115, USA

**Keywords:** precision medicine, pharmacogenetics, polymorphisms, pharmacogenomics, perioperative care

## Abstract

**Background:** Precision medicine represents an evolving approach to improve treatment efficacy by modifying it to individual patient’s gene variation. Pharmacogenetics, an applicable branch of precision medicine, identifies patient’s predisposing genotypes that alter the clinical outcome of the drug, hence preventing serious adverse drug reactions. Pharmacogenetics has been extensively applied to various fields of medicine, but in the field of anesthesiology and preoperative medicine, it has been unexploited. Although the US Food and Drug Administration (FDA) has a table of pharmacogenomics biomarkers and pharmacogenetics, this table only includes general side effects of the included drugs. Thus, the existing FDA table offers limited information on genetic variations that may increase drug side effects. Aims: The purpose of this paper is to provide a web-based pharmacogenomics search tool composed of a comprehensive list of medications that have pharmacogenetic relevance to perioperative medicine that might also have application in other fields of medicine. **Method:** For this investigation, the FDA table of pharmacogenomics biomarkers in drug labeling was utilized as an in-depth of drugs to construct our pharmacogenetics drug table. We performed a literature search for drug–gene interactions using the unique list of drugs in the FDA table. Publications containing the drug–gene interactions were identified and reviewed. Additional drugs and extracted gene-interactions in the identified publications were added to the constructed drug table. **Result:** Our tool provides a comprehensive pharmacogenetic drug table including 258 drugs with a total of 461 drug–gene interactions and their corresponding gene variations that might cause modifications in drug efficacy, pharmacokinetics, pharmacodynamics and adverse reactions. This tool is freely accessible online and can be applied as a web-based search instrument for drug–gene interactions in different fields of medicine, including perioperative medicine. **Conclusion:** In this research, we collected drug–gene interactions in a web-based searchable tool that could be used by physicians to expand their field knowledge in pharmacogenetics and facilitate their clinical decision making. This precision medicine tool could further serve in establishing a comprehensive perioperative pharmacogenomics database that also includes different fields of medicine that could influence the outcome of perioperative medicine.

## 1. Introduction

Precision medicine is an evolving approach to enhance treatment efficacy by tailoring it to the individual patient’s gene variation and environment [1]. One of the biggest advantages to such a method is the prevention of serious adverse drug reactions by identifying patient’s predisposing genotypes that alter the clinical outcome of the drug [2]. The broad concept of precision medicine includes an important applicable branch known as pharmacogenetics, the study of how patients’ genetic variation can alter their response to certain drugs. Genetic mutations can alter cellular transporters and channels involved in drug mechanism of action or the enzymes involved in their activation or degradation [3]. The benefits of pharmacogenetics implementation in the routine clinical setting are to assist in the precision of dosage, drug efficacy, mechanism of drug action, and avoidance of adverse side effects [4]. It could also play an important role in distinguishing responders and non-responders to drugs.

One of the early discoveries that helped to form the foundation of pharmacogenetics arose from the field of anesthesiology. Polymorphisms in the RYR1 gene have been shown to result in reduced activity of the enzyme cholinesterase and predispose certain individuals to life-threatening prolonged apnea after succinylcholine administration [5]. The completion of the Human Genome Project (National Institutes of Health, 2018), as well as the mapping of the 1.42 million additional single nucleotide polymorphisms (SNPs) between the years 2000 and 2003 [6], paved the way for the most commonly used research techniques today in pharmacogenetics. Typically, studies have tended towards either focusing on a handful of genes in what is referred to as the candidate gene approach or have preferred to cast a wider net via a genome-wide association study in which a much larger number of markers can be investigated at once [7]. The modern-day trend of implementing pharmacogenetics within the hospital system has also shifted with advancements in the field. Recognized institutions such as Vanderbilt University Medical Center [8] and St. Jude Children’s Research Hospital [9] have incorporated preemptive genetic testing as part of their routine patient care. Data from such institutions led to the issuing of a 2014 Department of Health & Human Services (DHHS) manual directed on how to implement preemptive pharmacogenetics [4]. 

Pharmacogenetics has been extensively applied to various fields of medicine. For example, in cardiology, allelic variations that involve metabolism of drugs such as statins, beta-blockers, and clopidogrel are being investigated [10], and in psychiatry, CYP2D6 alleles are being explored for their association with rates of tardive dyskinesia [11]. 

A patient’s response to a treatment depends on a variety of factors, which include the concomitant medications and genetic factors. Therefore, we consider the significance of having the most recent information of the pharmacogenomics of a variety of medications in which a perioperative patient may be on [1]. When it comes to pharmacogenetics, the field of anesthesiology and preoperative medicine remains a relatively untapped source of knowledge. Globally, almost 234 million people go through surgical procedures per year. Of these, 7 million patients experience a post-surgical adverse effect and 1 million patients die post-operatively each year [12]. Studies and their systematic review estimated a perioperative mortality rate of 1176 per million between 1990–2000. Pharmacogenetics could be one of the contributing factors leading to anesthesia and perioperative related deaths [13]. 

Most of the studies in the field of anesthesiology have focused only on the pharmacogenetics information of drugs that are used intraoperatively such as volatile anesthetic agents and muscle relaxants like halothane, desflurane, sevoflurane, isoflurane, and succinylcholine [12,14]. These studies have shown how genetic variations can provoke a hypermetabolic and potentially fatal state known as malignant hyperthermia (MH) in certain patients. However, in reality, anesthesiologists are usually challenged with managing the multimodal pharmacotherapy of each patient perioperatively. These engagements include, but are not limited to, titration of medications for pain, anesthesia induction and maintenance, muscle relaxation, antiemetic, anticoagulants, and beta-blockers. Different enzymes are involved in the mechanism of actions and metabolism of all these drugs. Genetic variations among individual patients play an important role in drug efficacy, toxicity, and interactions with other medications. Hence, having pharmacogenomics information unique to each patient before surgery would be a valuable tool to the anesthesiologists and other specialists for providing a tailored and optimized perioperative care to patients.

The purpose of this paper is to serve as a guideline that will assist in patient care with an emphasis on precision in perioperative medicine by providing a comprehensive list of medications that have pharmacogenetic relevance to perioperative medicine, i.e., personalized treatment in general anesthesia and surgery. Some of these drugs have a clinical use outside the perioperative setting and are also relevant to the perioperative setting due to their benefits in patient care prior to or following surgery. We aimed to provide a comprehensive list of drugs, along with their respective adverse effects, that have been shown to have variable pharmacokinetic/pharmacodynamics (PK/PD) due to the presence of metabolizing enzyme genetic polymorphisms, mutations, deletions, and treatment relevant biomarkers. Our list of drugs with the implication in precision medicine was compiled using a broad literature search revolving around the perioperative period and includes, but is not limited to, medications with US Food and Drug Administration (FDA) approved drugs with labeling for pharmacogenetic biomarkers [15]. 

## 2. Aims

This study is aimed at providing a web-based search tool where users have access to our in-depth list of medications that span across all fields of medicine. This application also allows users to search and filter for specific gene mutations to view the possible adverse effect among different drugs. The drugs are further classified into their respective specialty and perioperative stage relevance. The corresponding pharmacogenetic adverse effect for each specific polymorphism has also been collected with particular attention to certain drug reactions that might occur in more than one genetic variation. This tool could further serve in establishing a comprehensive perioperative pharmacogenomics database, which also includes different fields of medicine that could influence the outcome of perioperative medicine. By virtue of the tool, physicians can search for any drug and view all possible adverse effects as the result of genetic variations. 

## 3. Methods

The U.S Food and Drug Administration has been a strong advocate for pharmacogenomics since its inception and has provided drug labels containing genetic information since 2009 [16]. For this project the FDA table of pharmacogenomics biomarkers in drug labeling (hereafter the FDA table) [15] was completely reviewed and was utilized as a comprehensive list of drugs to construct our pharmacogenetic drug table. The FDA table contains the common side effect of the drugs and their corresponding biomarkers listed in the label. However, this table offers limited information as to the specific effects of the drugs among patients with genetic variations, and it mostly states the general side effects of the included drugs. We used the FDA table as a data resource for an in-depth list of drugs and their biomarkers and hence searched the literature for any genetic variation and related adverse effect of these drugs. The review was conducted between January 2017 and September 2019 using the drug name, as identified in the FDA table of pharmacogenomics, as well as the terms of polymorphisms, genetics, drug–gene interaction, and the specific identified mutations. Publications dating back to the year 2000 were included to maintain the most up-to-date information.

We reviewed all the labels for the New Drug Application (NDA) in the FDA Pharmacogenomic Biomarkers table. We searched for the following keywords in each drug label file: “pharmacogenomics”, “genetic”, “clinical pharmacology”, “warnings and dosage and administration”, “population subgroups”, and “use in a specific population”. We excluded drugs that did not have any specific drug–gene information on their labels. For those drugs with drug–gene information on their label, we conducted a literature search for publications that discuss that drug–gene interaction’s adverse effect. In most cases, the publication that we found discussed additional polymorphism (SNP) or genes for any specific drug and also referred to other drug–gene interactions; all the additional information was extracted and included in our drug table. Furthermore, if there were more than one publication describing each FDA table drug–gene interactions, all were fully reviewed, and additional information was included in our final pharmacogenetic table. As an example, the drug clopidogrel is listed in the FDA table with the corresponding biomarker of *CYP2C19*. We searched the above-mentioned keywords in the FDA drug label text, in which some of them could be found. Given we found specific drug–gene information on the FDA drug labels, we conducted a literature search for publications that discuss the corresponding drug–gene interactions’ adverse effect. After we reviewed the relevant publications, we added the additional information extracted from those papers to our main drug–gene table. For instance, in the case of clopidogrel, we added additional genes such as *CYP2B6*, or *ABCB1* and their polymorphisms as well as additional adverse effects that was not listed in the FDA table. For each additional piece of information extracted, we inserted the source of the information including the paper’s link, title, authors, and year of publication. In our table, polymorphism column comprises different forms of genetic variability such as alleles, phenotypes, haplotypes, and rsIDs.

At the time of this study, the FDA table contained 362 drug–gene interactions. Therefore, we performed a literature search for drug–gene interactions in a unique list of drugs in the FDA table. Publications containing the drug–gene interactions were identified and reviewed. Additional drugs and their extracted gene interactions in the identified publications were added to the constructed drug table. The search results that were added to the pharmacogenetic drug table included drug name, publication reference, author(s), year of publication, genetic mutation/polymorphism, the applicable field of medicine perioperative classification (preoperative, intraoperative, or postoperative), and adverse effects. Our pharmacogenetics table focuses on the list of drugs that were found to have adverse reactions on patients with specific genetic mutations that alter the drug’s pharmacokinetics and pharmacodynamics. 

To provide a web-based search tool, our final pharmacogenetics table’s data was loaded as a JavaScript Object that was later fed into a DataTable Object (https://datatables.net/) version 1.10.12. To have a responsive table and webpage, i.e., a webpage that could rearrange and adjust appropriately based on the screen size, we used bootstrap’s grid system version 4.0.0-alpha (https://getbootstrap.com/). The packages required jQuerry (https://jquery.com/), a fast and feature-rich JavaScript library. All packages and their dependencies were loaded on the client using their appropriate Content Delivery Network (CDN) address. Since all the technologies used here were based on JavaScript, the application would be a front-end solution in which users interact directly with it. All the filtering and rendering were performed by the client device.

## 4. Result

### 4.1. Overview of the Tool

**Figure 1:**
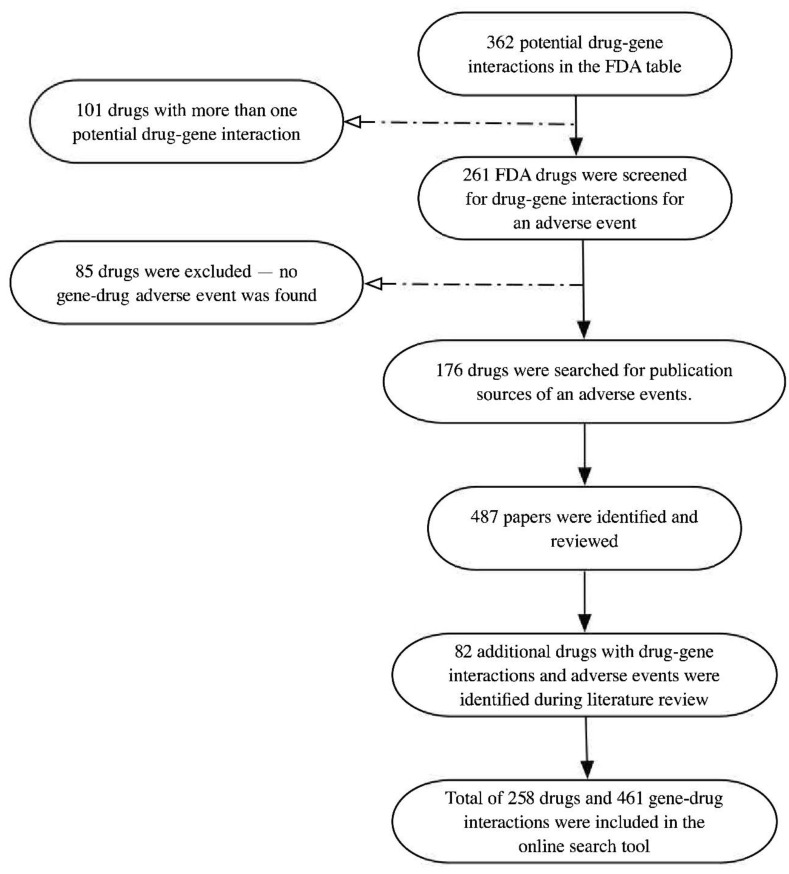
Flow chart of the selection of drugs using the Table of Pharmacogenomic Biomarkers in Drug Labeling by Food and Drug Administration (FDA) and literature review process to identify the drug–gene interactions and potential adverse effects in the different fields of medicine, including perioperative medicine.

We constructed a comprehensive pharmacogenetics table with a total of 258 drugs and their corresponding gene variants or mutations (N = 461) that might cause modifications in drug efficacy, PK/PD and adverse reactions. The data table is accessible on https://pharmacogenomics.github.io/pharmacogenetics/. As mentioned in the method section, we used the FDA table [15] as our main source of data for drug–gene interactions. The process of drug selection is illustrated in Figure 1. 

### 4.2. Frequency of Drug–Gene Interactions

At the time of this study, the FDA table contained entries including 362 drug–gene interactions with 101 entries related to the same drugs and 85 drug–gene combinations with no adverse effect. Hence, we performed a literature search for each of the remaining 176 drug–gene interactions for evidence on any additional information on drug–gene interactions. Reviewing the identified papers, we collected the report on additional drug–gene interactions as well as any drugs that were not stated in the FDA table and included them in our pharmacogenetics table in addition to the information in the FDA table. In total, 487 publications were reviewed, and we found 82 additional drugs that were not listed in the FDA table but have important information that may lead to adverse reactions in patients with specific gene variations. Our final table consists of 258 drugs and 461 drug–gene interactions (414 unique genes). In total, 68.22 (176/258) of the included drugs were listed in the FDA table and the remaining 31.18 (82/258) were found using the literature review.

Table 1 lists the percentages of data for drug–gene interactions that were found from our main table for each different field of medical specialties. The top seven fields were oncology, infectious disease, cardiology, psychiatry, anesthesiology, neurology, and hematology. Some drugs applied to more than one field. For example, celecoxib, warfarin, and clopidogrel were listed in both cardiology and hematology, or diazepam was listed in both neurology and anesthesiology. 

### 4.3. Frequencies by Speciality

In Table 2, for each medical specialty, we provided the percentage of drugs with available pharmacogenetic information and the involved genes that their variations cause drugs’ adverse reactions. Such drug–gene interactions were most common in oncology and least common in the field of ophthalmology. Some genes were common across different fields (e.g., *HLA-B* in neurology and infectious disease). 

### 4.4. Pharmacogenes

**Figure 2:**
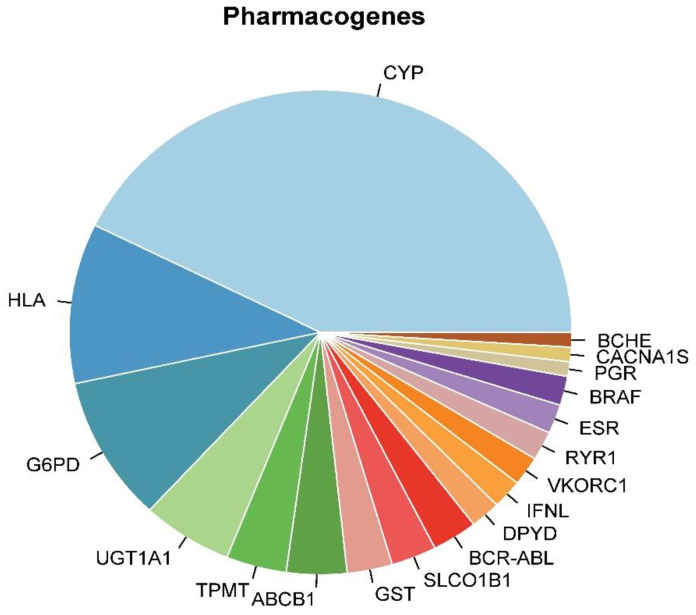
Pharmacogenes’ relative contributions to adverse events. CYP mutations include CYP1A2, CYP2A6, CYP2B6, CYP2CP9, CYP2C8, CYP2C19, CYP2D6, CYP2E1, CYP3A4, CYP3A5, and CYP4A11.

Table 3 describes the percentage that each pharmacogene contributes to a drug–gene adverse reaction, and Figure 2 illustrates the relative comparison of each pharmacogene. As it shows, Cytochrome P450 (CYP), followed by HLA, G6PD, and UGT1A1 gene variations cause the most adverse reactions among all drugs. 

**Figure 3:**
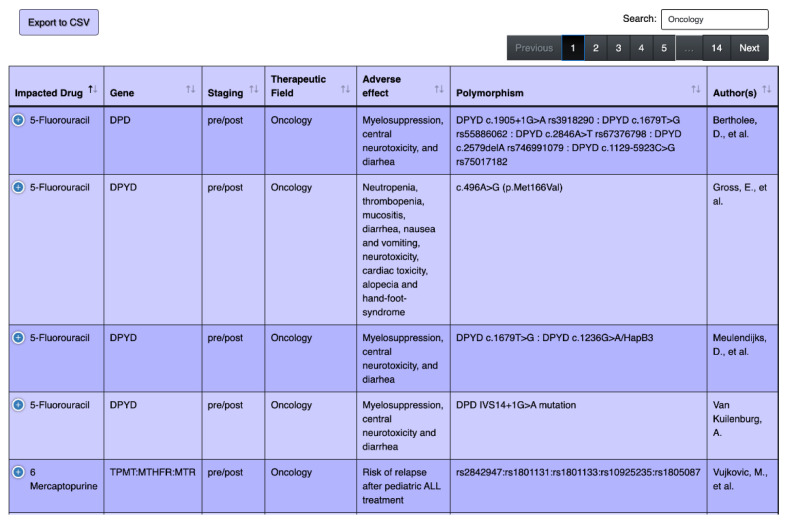
Screenshots of the web-based search tool of pharmacogenetics table, searching for drugs related to the field of oncology. The background color is the same as the actual website, color variation is for row separation purpose.

**Figure 4:**
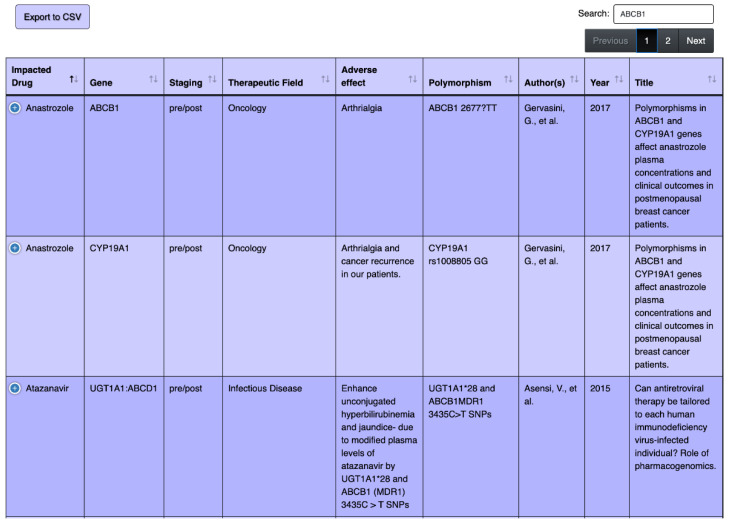
Screenshots of the web-based search tool of pharmacogenetics table, searching for drugs that have adverse effects on the ABCB1 gene. The background color is the same as the actual website, color variation is for row separation purpose.

**Figure 5:**
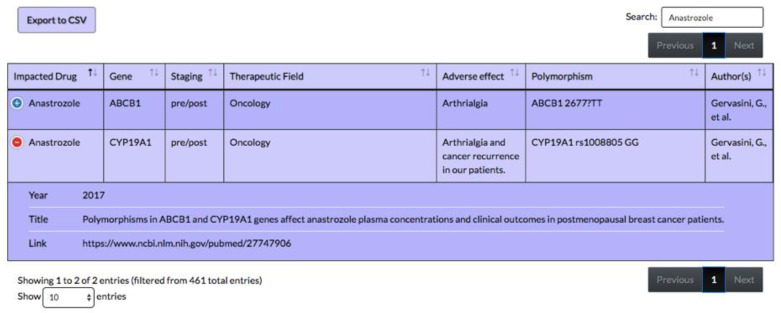
Screenshots of the web-based search tool of pharmacogenetics table, searching for adverse effects of anastrozole on different genes. Expansion of cells using the plus sign provides the source and year of publication of the reference article. The background color is the same as the actual website, color variation is for row separation purpose.

Figure 3, Figure 4 and Figure 5 demonstrate the screenshots of our web-based pharmacogenetics table. As we illustrated in Figure 3, a user can search for any specific field of medicine and view all drug–gene interactions, e.g., oncology as an example in this figure.

Moreover, a user can search for a specific gene (for example gene ABCB1 in Figure 4) and view all corresponding drugs that interact with that gene. Furthermore, a user can also search by the name of a drug, e.g., anastrozole demonstrated in the screenshot in Figure 5. More detailed instruction on how to use the table was provided on the webpage (https://pharmacogenomics.github.io/pharmacogenetics/).

### 4.5. Intraoperative Precision Medicine: Anesthesiology

We found 20 anesthetic drugs composed of 7.75% of pharmacogenes. Inhaled anesthetic and intravenous opioid analgesic agents were the most common anesthetic medications that might induce a drug–gene interaction and potentially a subsequent intraoperative adverse event [12,17,18]. Genes that were mostly interacting with anesthetic drugs were *CYP2D6*, *RYR1*, *CYP3A4*, *BChE*, and G6PD [19].

## 5. Discussion

This study provides a web-based tool with a comprehensive list of drugs, along with their respective adverse effects, that were shown to have variable pharmacokinetic/pharmacodynamics (PK/PD) due to the presence of metabolizing enzyme genetic polymorphisms, mutations, deletions, and treatment relevant biomarkers. Although we also provided additional important common non-anesthetics drugs that might perioperatively influence the outcome of anesthesia, the focus of this research was the pharmacogenetics of anesthetic drugs. Hence, anesthesiologists and physicians in other fields can utilize our web-tool as a quick and accessible resource to obtain information on drug–gene interactions to prevent perioperative related morbidity and mortality.

Almost one out of four outpatients receive one or more drugs with pharmacogenomic biomarker information in its label [20]. Each drug-biomarker pair could have different meanings and implications for clinicians and patients. Not every drug with a biomarker label requires genetic testing before prescribing. The biomarker could be just listed on the label to define the corresponding drug’s mechanism of action, or in some cases, it could represent decreased efficacy or increased toxicity among the biomarker-based subpopulation. Therefore, these labels with pharmacogenomics information are not fully relevant to personalized medicine [20,21]. Previous analysis of the FDA table showed that only 12% of drugs licensed in the period 1998–2012 had labels with pharmacogenetics information [22]. The inclusion of this information had no direct clinical usage, and only 14 drugs required pharmacogenetics testing before prescribing the drugs [22]. For this research, we mostly listed drug-biomarker pairs that cause an adverse effect in a specific population with biomarker-based genetic variation. In other words, genetic testing should be required in most of the drugs listed in our table.

### 5.1. Intraoperative Precision Medicine: Anesthesiology

Some of the most important pharmacogenomic adverse events pertinent to anesthesiology are prolonged apnea after succinylcholine administration due to deficiency or polymorphisms of pseudocholinesterase (PChE) or butyrylcholinesterase (BChE) and malignant hyperthermia after administration of volatile anesthetic agents due to mutation in the genes encoding the ryanodine receptor (RYR1) gene and calcium voltage-gated channels dihydropyridine receptor (DHPR, CACNA1S) [23,24]. Halothane, along with other volatile anesthetic agents such as desflurane, sevoflurane, and isoflurane as well as succinylcholine have been shown to provoke a hypermetabolic and potentially fatal state of malignant hyperthermia in certain patients [14]. With the use of pharmacogenetics, patients could be screened for an increased probability of negative outcomes. Mutations mostly involving the RYR1 gene and the CACNA1S gene (to a lesser extent) [25] lead to an uncontrolled release of calcium from the sarcoplasmic reticulum responsible for the characteristic signs and symptoms of MH [26].

Reactions to anesthetic medications have a wide variability of responses owing to the high degree of allelic variants that code for metabolizing enzymes, drug receptors, nuclear receptors, and transport proteins and receptors [12]. Thus, both sub and supratherapeutic drug levels can lead to serious consequences such as those observed in postoperative pain management with the use of opioids such as codeine or morphine [27]. Codeine is converted to morphine, the active metabolite, which induces the necessary anesthesia [28]. Morphine toxicity, however, can result in respiratory depression and may even lead to death, if not managed appropriately. Polymorphisms in CYP2D6 can affect the rate of codeine conversion to morphine, which can lead to either decreased amounts of active metabolite or toxicity at standard dosage [28]. CYP2D6*1*1 × N and CYP2D6*1/*2 × N alleles are denoted to be ultra-rapid metabolizers and might increase the risk of respiratory depression. On the other hand, carrying the poor metabolizer (PM) CYP2D6*4/4, CYP2D6*4/5, CYP2D*5/*5, CYP2D*4/6 could decrease the alleviation of pain due to less active metabolite formation [29]. These unwanted side effects highlight the importance of further incorporation of pharmacogenetics into anesthesiology.

The most common enzymatic disorder of red blood cells is G6PD deficiency. Patients with G6PD deficiency are susceptible to oxidative stresses from certain drugs including those used intraoperatively. Benzocaine, lidocaine, prilocaine, and chloroprocaine can all cause drug-induced methemoglobinemia among G6PD deficiency patients [30,31].

### 5.2. Perioperative Precision Medicine: Fields Other Than Anesthesiology

While the focus of this research was on perioperative pharmacogenomics, we expanded our search into other fields of medicine. This expansion was owing to the interference of non-anesthetic drug interactions in perioperative medicine and the fact that their inherited genetic variations can also contribute to the patients’ perioperative outcome. Hence, it is imperative to discuss the pharmacogenomics of different drug classes related to patients undergoing surgery.

For instance, many drugs could be substrate inducers or inhibitors of CYP2D6. Some examples are psychiatric medications, such as tricyclic antidepressants (TCAs), selective serotonin reuptake inhibitors (SSRIs), serotonin-norepinephrine reuptake inhibitors (SNRI), and antipsychotics. In other fields of medicine, other drugs include beta-blockers, antiarrhythmic, antiepileptic, antiemetic, analgesic, calcium channel blockers, and antihistamines [32,33]. Depending on the genetic variation of the CYP2D6 enzyme, a patient may experience mild to severe adverse effects when taking any of these drugs. In some cases, patients with the ultra-metabolizer variation of *CYP2D6* could experience treatment failure and not be responding to medication while those with poor metabolizer could experience severe life-threatening reactions such as heart failure, cerebrovascular events, fatal respiratory depression, and even death [34,35].

For instance, in patients with CYP2D6 poor metabolizer enzyme, beta-blockers such as metoprolol and propranolol can cause a rash, bradyarrhythmia, shortness of breath, hypotension and heart failure [34]. Therefore, to prevent or lessen these adverse effects, anesthesiologists and other physicians should consider drugs’ dose adjustments based on patients’ CYP2D6 status especially during the perioperative period.

In this research, we tried to construct a web-based comprehensive pharmacogenetics tool where clinicians can easily search for any specific drug and see if there is any associated drug–gene adverse effect. Table 1 shows the highest proportion (28%) of our table’s pharmacogenetics information belongs to the field of oncology. Another study that focused only on the FDA table showed 29% of the FDA drug–gene pairs had a genetic biomarker testing recommendation on their labels. Most of the labeled drugs were found in the field of oncology as compared with all the other fields of medicine (98/158 [62%] vs. 19/158 [12%]; *p* < 0.001). Higher risk of toxicity among oncology drugs was the main reason for pharmacogenomic labeling (87/158 [55%]) [21]. Therefore, the field of oncology has discovered the highest amount of pharmacogenetics information compared to the other fields of medicine.

Figure 2 illustrates the portions of pharmacogenetics’ contributions to most commonly found genes. The top nine most common genes were *CYP*, *UGT1A1*, *BCR-ABL*, *G6PD*, *RYR1*, *BRAF*, *HLA*, *BCHE* and *DPYD*. These genes impact drug-related pharmacokinetics by controlling the functionality of target receptors (RYR1 gene), drug metabolism (*CYP*, *G6PD*, *BCHE*, *DPYD*, *UGT1A1*), drug transport, neurotransmitters or immune components (*HLA*), and proto-oncogene genes (*BRAF*, *BCR-ABL*), where all of these genes may also be part of a drug’s mechanism of action. Thus, just as anesthetic drugs, non-anesthetic drugs in all different fields should be of paramount importance to the anesthesiologists. Some of the most common non-anesthetic medications that are commonly used perioperatively include opioid and non-opioid analgesic, benzodiazepine, antiemetic, anticoagulants and beta-blockers. These perioperative medications and their pharmacogenomics were fully reviewed in several studies [23,24]. However, other drugs from different fields, e.g., oncology or psychiatry, have not been studied in the field of perioperative pharmacogenomics. If a cancer patient requires a surgical procedure, all involving physicians should be aware of the type of chemotherapeutic drugs that the patient is taking before surgery and check for any adverse reaction due to their pharmacogenetics. According to Table 1, the ABCB1 gene was one of the most common genes in oncology that are associated with adverse effect. This gene encodes an important efflux transporter of P-glycoprotein (P-gp), also known as multidrug resistance-associated protein 1 (MDR1), at the blood–brain barrier that controls the central nervous system rate of drug absorption and exposure. Genetic polymorphism in the ABCB1 gene has been associated with significant changes in CNS pharmacokinetics of opioid drugs by either limiting their uptake or increasing their clearance form brain into the blood [36]. Therefore, even though this specific gene is mostly associated with the pharmacokinetics of oncology-related drugs such as anastrozole, doxorubicin, everolimus, and cyclosporine [37], its adverse drug–gene interaction can manifest at any point in the perioperative period. Therefore, a comprehensive understanding of pharmacogenomics will allow anesthesiologists and other physicians for more individually tailored perioperative drug administration that will prevent any adverse events.

### 5.3. Future Directions

Medicine is a constantly evolving science with new drugs constantly developed as well as new applications of existing drugs being discovered. Unknown drug interactions of existing medicines are likely to exist as more patients are prescribed the drugs for both existing as well as new uses. The more information that is divulged to the medical community, the more likely that these adverse drug interactions are discovered. Thus, this research is limited by the existing data that were consolidated into a more user-friendly application. Further clinical studies are recommended to examine the efficacy and burden of these drug–gene interactions in the general population and to establish programs that could provide preventive instructions to clinicians. Programs such as Trans-Omics for Precision Medicine (TOPMed) have been initiated with the focus on integrating metagenomic data to improve the prevention and treatment of heart, lung, blood, and sleep disorders [38].

When it comes to clinical decision-making, pharmacogenomics should be considered as another important factor that could alter the risk of adverse drug effects and subsequent outcomes of patients in need of surgery. Even though the field of pharmacogenomics has been growing and more genetic polymorphisms have been discovered, preoperative pharmacogenomic testing is still underdeveloped in the US and many of the insurance companies do not cover such services [23]. Establishing perioperative pharmacogenomics requires convincing clinical evidence generated by conducting large-scale randomized controlled clinical trials to prove the efficacy and cost-effectiveness when pharmacogenomics-based treatments are utilized.

This study provided an online search tool for drug–gene interactions and potential adverse reactions that can be applicable in perioperative medicine and their overlap with a wide range of other fields of specialties, as shown in Table 2. Thus, we did not eliminate the data that could be relevant to other specialties outside anesthesiology. As such, future research is needed to test the applicability of this tool and other similar ones on the enhancement of clinical care in perioperative medicine and across other fields of medicine.

One of the major implications of the web-based tool presented in this study is to enhance the clinical care of patients using Electronic Health Record (EHR) systems and predictive science. Researchers have already advocated leveraging of the EHR to enhance EHR-based precision medicine by associating genotyping information [39]. Although each health care system has a unique EHR system, almost all EHR systems include diagnostic as well as prescribed medications. We argue that if some genetic data are also included in some EHR, then this tool could be merged with the EHR or could be used jointly with the EHR. Such efforts may enhance the utility and the impact of our web-based search tool.

## 6. Conclusions

We provided a web-based search tool that includes an in-depth list of drug–gene interactions applicable by physicians to expand their field knowledge in pharmacogenetics of adverse drug reactions in perioperative medicine and facilitate their clinical decision making across the overlapping fields of medicine with perioperative medicine. This tool could further serve in establishing a comprehensive perioperative pharmacogenomics database, which also includes different fields of medicine influencing the outcome of perioperative medicine. By virtue of the tool, physicians can search for any drug and view all possible adverse effects as the result of genetic variations.

## Figures and Tables

**Table 1:** Most common genes associated with adverse effect of drugs among different fields of medicine.

Field of Medicine	Most Common Gene Associated with Adverse Effect in an Interaction with Drugs
Oncology	ABCB1, BCR-ABL, BRAF, CYP3A4, DPD, ESR, PGR, TPMT, UGT1A1
Infectious Disease	CYP2C8, CYP2C19, G6PD, HLA-B, HLA-A, IFNL3/ IFNL4, UGT1A1
Cardiology	ABCG2, CYP2D6, SLCO1B1
Psychiatry	CYP1A2, CYP2C19, CYP2D6
Anesthesiology	BCHE, CYP2D6, CYP3A4, G6PD, RYR1
Neurology	CYP2C19, CYP2D6, GST M1, HLA-B
Hematology	CYP2C9, CYP2C19, VKORC1

**Table 2:** Contributions of fields of medicine to the pharmacogenetic information in the collected data.

Field of Medicine	% Drugs with Pharmacogenetic Information
Oncology	27.88%
Infectious Disease	14.95%
Cardiology	13.94%
Psychiatry	9.29%
Anesthesiology	7.75%
Neurology	6.67%
Hematology	6.06%
Gastroenterology	4.85%
Rheumatology	3.64%
Endocrinology	2.22%
Dermatology	0.81%
Gynecology	0.81%
Pulmonary	0.81%
Urology	0.61%
Genetic Medicine	0.40%
Ophthalmology	0.20%

**Table 3:** Pharmacogenetics’ contributions for the most commonly found genes.

Genes	% Drug–Gene Interactions
CYP	44%
HLA	11%
G6PD	10%
UGT1A1	6%
TPMT	4%
ABCB1	4%
GST	3%
SLCO1B1	3%
BCR-ABL	3%
DPYD	2%
IFNL	2%
VKORC1	2%
RYR1	2%
ESR	2%
BRAF	2%
PGR	1%
CACNA1S	1%
BCHE	1%
